# Fluid resuscitation strategy in patients with placenta previa accreta: a retrospective study

**DOI:** 10.3389/fmed.2024.1454067

**Published:** 2024-09-24

**Authors:** Fan Zhou, Na Liu, Guiqiong Huang, Haiyan Yu, Xiaodong Wang

**Affiliations:** ^1^Department of Obstetrics and Gynecology, West China Second University Hospital, Sichuan University, Chengdu, Sichuan, China; ^2^Key Laboratory of Birth Defects and Related Diseases of Women and Children (Sichuan University), Ministry of Education, Chengdu, Sichuan, China; ^3^Department of Gynecology, The First People’s Hospital of Yunnan Province, Kunming, Yunnan, China

**Keywords:** placenta previa, placenta accrete, hemodynamic indicator, fluid resuscitation, post-partum hemorrhage

## Abstract

**Objectives:**

Obstetric hemorrhage is the leading cause of maternal death worldwide. Placenta previa accreta is one of the major direct causes of postpartum hemorrhage, accounting for two-thirds of obstetric hemorrhage cases. Fluid resuscitation is a life-saving procedure for patients suffering from massive hemorrhage. This study aims at evaluating the risk factors of massive hemorrhage and appropriate fluid resuscitation strategy in patients with placenta previa accreta.

**Methods:**

This study retrospectively analyzed the risk factors for massive hemorrhage, clinical characteristics, and perinatal outcomes of patients with placenta previa accreta. Maternal noninvasively evaluated hemodynamic indicators, including maternal heart rate, systolic blood pressure (SBP), diastolic blood pressure (DBP), mean arterial pressure (MAP), and shock index, were collected and analyzed at nine time points, from the administration of anesthesia until the end of procedures, in patients diagnosed with placenta previa accreta and receiving different fluid supply volumes.

**Results:**

Complicated with placenta increta/percreta and gestational age of delivery later than 37 weeks are two independent risk factors of massive hemorrhage in patients with placenta previa accreta. A total of 62.27% (170/273) patients diagnosed with placenta increta/percreta had massive hemorrhage, significantly higher than those diagnosed with placenta previa accreta (5.88%, 6/102). Patients delivered after 37 weeks of gestation had significantly higher ratios (86.84%, 99/114) of massive hemorrhage compared with those delivered between 36 and 36^+6^ weeks of gestation (35.39%, 63/178). Maternal SBP, DBP, and MAP started to decrease immediately after the baby was delivered and reached a relatively stable trough state at 15–30 min after delivery. No statistical differences were found in hemodynamic indicators, the occurrence of hypotension, or in-hospital days after the procedure among the transfusion volumes < 30 ml/kg, 30–80 ml/kg, and ≥ 80 ml/kg groups.

**Conclusion:**

Patients with a suspected diagnosis of placenta previa accreta should plan to deliver before 37 weeks of gestation. The ability to identify concurrent placenta increta/percreta should be improved to schedule a reasonably rapid perioperative plan. Restrictive fluid resuscitation could achieve good effects in maintaining hemodynamic stability in patients with placenta previa accreta. A time period of 15–30 min after delivery is the critical stage for fluid resuscitation.

## Introduction

Obstetric hemorrhage, accounting for 27.1% of total maternal deaths, was the leading direct cause of maternal death worldwide between 2003 and 2009 according to a World Health Organization (WHO) systematic analysis (1). Obstetric hemorrhage was also the leading cause of maternal death in China from 1990 to 2019, in spite of the fact that the national maternal mortality rate resulting from obstetric hemorrhage had dropped by 91.7% in the year 2019 (3.0 per 100,000 live births), compared with the year 1990 (36.3 per 100,000 live births) (2). More than two-thirds of hemorrhages leading to maternal deaths occur in the postpartum period and are known as postpartum hemorrhage (PPH) (1). Placenta accreta spectrum (PAS) and placenta previa are two significant risk factors for PPH (3, 4). The incidence of placenta previa accreta in China has increased exponentially as a result of rising rates of prior cesarean deliveries or uterine surgery and the three-child policy (3, 4).

Placenta previa refers to pregnant women with a placental edge less than 20 mm from the internal os or covering the os, shown by imageological examination after 28 weeks of gestation (5). PAS is separated into the following three categories: patients with placenta villi adhering to the myometrium, referred to as placenta accreta; patients with placenta villi invading the myometrium, referred to as placenta increta; and patients with placenta villi invading the full thickness of the myometrium, referred to as placenta percreta (4–7). The abnormally invasive placenta does not separate spontaneously after delivery and might result in life-threatening bleeding during the removal procedure. The reported incidence of PAS varied from 0.79 per 1,000 births to 3.11 per 1,000 births (8). Patients with placenta previa accreta met the diagnostic criteria for both placenta previa and PAS simultaneously. Optimal management of patients with abnormally lying or invasive placenta requires both accurate antenatal imageological examination and a reasonable and rapid perinatal management strategy. Most researchers have focused on the risk factors, optimal gestational age for delivery, uterotonic treatment, and hemostatic techniques of placenta previa and PAS (5, 9). Less attention has been paid to fluid resuscitation during the perioperative period in this group of pregnancies with a high risk of massive obstetric hemorrhage.

Patient blood management aims to maintain hemoglobin concentration, optimize hemostasis, and minimize blood loss to improve patient outcomes after surgery (10). Pregnant women have hemodynamic characteristics such as high hypervolemia, high hyperdynamic circulation, and high oxygen consumption (11, 12). The fraction of cardiac output distributed to the uterine circulation increased almost linearly from 5.6% at 22 weeks of gestation to 11.7% at 39 weeks of gestation (12), indicating that uterine bleeding would have a marked impact on patients’ hemodynamic stability. Fluid management is a crucial component of PPH treatment to maintain maternal hemodynamic stability and save the lives of parturients.

Restrictive fluid resuscitation policies apply a small amount of perioperative fluid infusion volume compared with liberal or traditional fluid resuscitation approaches (13). The fluid supply volume in restrictive policies should contain individual basal fluid requirements and blood loss volume in a 1:1 ratio, guided by an assessment of active hemorrhage in combination with changes in hemodynamics (14). Evidence indicates that restrictive fluid resuscitation is associated with a lower risk of adverse events than conventional fluid resuscitation in patients with both traumatic and non-traumatic hemorrhagic shock (15–17). Restrictive fluid resuscitation can avoid coagulation disorders caused by rapidly rising systolic blood pressure (SBP) (18). However, the status of the application of restrictive fluid resuscitation strategies in the perioperative period of patients with PPH caused by an abnormally lying or invasive placenta is still unknown. There is no consensus on how to perform fluid management in patients with placenta previa accreta to actively control obstetric hemorrhage and ensure the stability of hemodynamics. This study aims at retrospectively analyzing the real-world data of risk factors of massive hemorrhage and fluid resuscitation strategy in patients with placenta previa accreta, thus providing evidence for optimal perioperative fluid management.

## Materials and methods

### Participants inclusion

Pregnancies complicated with placenta previa and delivered in the West China Second University Hospital, Sichuan University, between January 2015 to June 2019 are included. The West China Second University Hospital is a specialist acute and critical treatment center for pregnant women and the puerperium, equipped with a multidisciplinary team with expertise in obstetric surgery and immediate access to blood products, adult intensive care unit and neonatal intensive care unit (NICU). This retrospective study involving human data collection was performed in accordance with the Declaration of Helsinki. Approval from the ethics committee of West China Second University Hospital, Sichuan University was obtained prior to patient enrollment.

Placenta previa is diagnosed according to the Royal College of Obstetricians and Gynecologists (RCOG) Green-top Guidelines (the placental edge is less than 20 mm from the internal os or covers the os with sonography scanning after 28 weeks of gestation) (5). Although transvaginal sonography is recommended and is superior to transabdominal and transperineal approaches for the diagnosis of placenta previa (1), most of the included patients were diagnosed using transabdominal and transperineal combined sonography because of the low acceptance of transvaginal operation in patients suspected of placenta previa.

The inclusion criteria were as follows: gestational weeks greater than 28 weeks; a previous cesarean section or myomectomy, and the placenta of the current pregnancy was attached to the uterine scar; placenta previa and PAS were suspected to be diagnosed by Doppler ultrasound and magnetic resonance imaging (MRI) during pregnancy. Placenta increta and placenta percreta were suspected antenatal in patients with disrupted integrity of the uterine endometrium and smooth muscle layers of the myometrium on imageological examination. PAS was diagnosed clinically during placenta separation and/or ascertained with placental histopathologic examination: placenta villi adhering to the myometrium without interposing decidual tissue were diagnosed as placenta accreta; placenta villi that invaded the myometrium were referred to as placenta increta, and placental villi that invaded the full thickness of the myometrium and crossed the uterine serosa were referred to as placenta percreta (4–7). The exclusion criteria were as follows: incomplete clinical data, multiple pregnancies, severe fetal malformations, and pregnancies complicated with severe internal medicine disease or hypertensive disorders (including participants manifested with moderate or severe anemia, cardiac dysfunction, which has not been corrected before delivery). All included patients provided informed consent for the risks associated with cesarean section, massive obstetric hemorrhage, and lower urinary tract damage related to an abnormally lying or invasive placenta and the possible need for blood transfusion.

For pregnancies antenatal suspected of placenta previa accreta, a selective delivery is recommended at 34–36 or 36–37 weeks of gestation in different consensus guidelines on placenta accreta spectrum (5, 8, 9, 19). We selected 37 weeks of gestation, the upper limit of recommended gestational weeks, as the cut-off value to analyze the risk of massive hemorrhage in women diagnosed with placenta previa accreta. Meanwhile, the recommended mode of delivery for patients suspected of placenta previa accreta is elective cesarean section (5). Surgical approach was determined by placenta position, depth of villous invasion, parametrial extension assessed by ultrasound and MRI before delivery, clinical symptoms of bleeding, as well as the visual assessment of the uterus by obstetricians during surgery (5). For women with placenta accreta limited in depth and the entire placental implantation area is accessible, uterus preserving surgery (partial myometrial resection and uterus repair) is performed by experienced obstetricians and appropriate expertise team. Surgical hemostatic technique, interventional radiology and intrauterine tamponade are used according to the actual condition to control intraoperative bleeding in women with uterus preserving surgery. Hysterectomy is quickly decided if conservative medical and surgical interventions prove ineffective. All cesarean section and hysterectomy were performed by experienced obstetrician and gynecologist in collaboration with senior anesthetist. A restrictive balanced crystalloid resuscitation, in a ratio of 1–2 mL of Ringer’s lactate per 1 mL blood loss, is used as initial perioperative fluid resuscitation and adjusted according to the context of clinical condition and estimated perioperative blood loss (10, 20).

### Data collection and analysis

We collected basic information and noninvasively evaluated hemodynamic indicators at nine time points from the administration of anesthesia until the end of the procedure. Blood loss volume is estimated by obstetrician, anesthesiologists and operating room nurse via collecting the numbers of medical gauzes immersed with blood, blood volume in the suction bottle, blood volume in the autologous cell salvage device, in combination with blood-gas analysis, blood routine test and vital signs measurement. The hemodynamics indicators include maternal heart rate (HR), systolic blood pressure (SBP), diastolic blood pressure (DBP), mean arterial pressure (MAP) and shock index (SI). The nine time points were recorded as the administration of anesthesia (TA), instantly after baby delivered (T0), 5 min after baby delivered (T1), 10 min after baby delivered (T2), 15 min after baby delivered (T3), 20 min after baby delivered (T4), 25 min after baby delivered (T5), 30 min after baby delivered (T6), and the end of procedures (T7). Hypotension refers to SBP < 90 mmHg, mean arterial pressure (MAP) < 65 mmHg, or blood pressure decreased by > 40 mmHg (21). PPH is defined as blood loss more than or equal to 1000 ml according to the American College of Obstetricians and Gynecologists (ACOG) guidelines (22). Severe PPH is defined as blood loss ≥ 1500 ml or the need for blood transfusion (23). Shock index (SI) was calculated by dividing the maternal heart rate by SBP. An SI of 0.85–0.90 may be an early sign of hypovolemia requiring appropriate intervention (24). In this study, we considered SI ≥ 1 at three continuous time points as hypovolemia. Neonatal mild asphyxia refers to 1 min or 5 min Apgar score between 3 and 7, while severe asphyxia refers to 1 min Apgar score < 3 or 5 min Apgar score < 5.

### Statistical analysis

SPSS 25.0 software (IBM, Armonk, New York, USA) was used to analyze the data. Measurement data with normal distribution is presented as mean ± standard deviation (SD), the comparison between two groups is performed with t-test, and the comparison among three or more groups is performed with analysis of variance (ANOVA). Multi-measurement data are analyzed with repeated measures ANOVA. For data failed in sphericity test, the statistical result is corrected with Greenhouse-Geisser. Measurement data with non-normal distribution is presented as median (interquartile range, IQR), the comparison between groups is performed with non-parametric test. Enumeration data is presented with frequency and ratio, the comparison between groups is performed with chi-square test. The risk factors of massive hemorrhage in patients with placenta previa accreta were analyzed with multivariate logistic regression analysis. The odds ratio (OR) and 95% confidence interval (CI) were presented. The significance level was set at bilateral α = 0.05.

## Results

### Basic information

**FIGURE 1:**
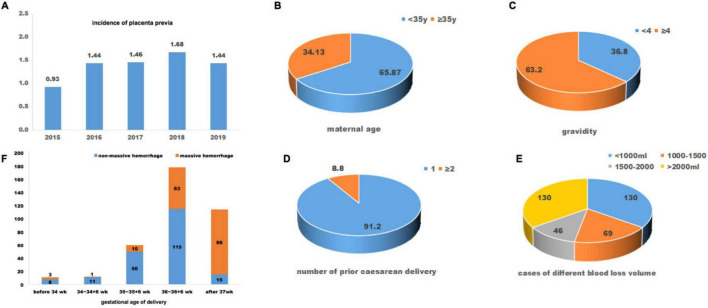
Baseline information of included patients with placenta preiva accreta. **(A)** incidence of placenta previa during year 2015 to 2019; maternal age **(B)**, gravidity **(C)**, number of prior caesarean delivery **(D)**, and cases of different blood loss volume **(E)** of included patients; **(F)**, gestational age at delivery and cases of massive hemorrhage.

During January 2015 to June 2019, a total of 53, 303 pregnancies delivered in the West China Second University Hospital, Sichuan University. In total, 708 patients (1.33%) were diagnosed with placenta previa. The incidence of placenta previa has increased over time, rising by years from 0.93% in 2015 to 1.44% in 2019 (Figure 1). According to the inclusion and exclusion criteria, 375 pregnancies with placenta previa accreta were included in this retrospective study. The mean maternal age and body mass index (BMI) of all included patients was 32.68 years and 26.41 kg/m^2^. Of them, 63.20% patients had four or more previous pregnancies. All included patients had prior cesarean section, and 8.8% of them had two or more previous cesarean deliveries (Figure 1). The interval time between the previous cesarean delivery and the current pregnancy ranged from one year to 18 years, and the median interval years was six years.

Intraoperative and postoperative blood loss volumes ranged from 250 ml to 12,000 ml. 65.33% (245/375) met the diagnostic criteria for PPH with a blood loss volume ≥ 1000 ml. 12.27% (46/375) of these patients had a blood loss volume between 1500–2000 ml, and 34.67% (130/375) had a blood loss volume ≥ 2000 ml; all these patients had severe PPH. Seventy-five patients underwent hysterectomy for uncontrolled uterine bleeding after conservative uterotonic treatment and hemostatic techniques. The gestational weeks of delivery in all included pregnancies were between 28 and 40^+6^ weeks, and the mean (SD) was 36.49 (1.14). Of these, 69.60% (261/375) patients delivered before 37 weeks of gestation, and 11 of 261 patients delivered during 28–33^+6^ weeks of gestation. Meanwhile, patients delivered after 37 weeks of gestation had significantly higher ratios (86.84%, 99/114) of massive hemorrhage compared with those delivered before 37 weeks of gestation (29.50%, 77/261) (*P* < 0.05, Figure 1). As for neonatal outcomes, 21.87% (82/375) of them had low birth weight (< 2500g), and the mean (SD) newborn birthweight was 2783.67 g (386.77 g). 7.20% (27/375) neonates had mild asphyxia (1 min or 5 min Apgar score between 3 and 7), and one neonate had severe asphyxia (1 min Apgar score < 3 or 5 min Apgar score < 5). 19.47% (73/375) of newborns were admitted to the NICU.

### Risk factors of massive obstetrical hemorrhage in patients with placenta previa accreta

All the included pregnancies were divided into two groups according to blood loss volume: blood loss volume ≥ 1500 ml in the massive hemorrhage group (n = 176), and blood loss volume < 1500 ml in the non-massive hemorrhage group (n = 199). BMI and parity were significantly higher in the massive hemorrhage group than in the non-massive hemorrhage group (*P* < 0.05). Maternal age, abortion history, number of previous cesarean sections, and hematology laboratory indicators demonstrated no statistical significance between the massive and non-massive hemorrhage groups (*P* > 0.05) (Table 1). Moreover, the ratio of placental increta/percreta and uterine artery embolization in the massive hemorrhage group was significantly higher than that in the non-massive hemorrhage group (*P* < 0.05) (Table 1). The gestational age at delivery averaged 37.11 weeks in the massive hemorrhage group compared with 35.95 weeks in the non-massive hemorrhage group (*P* < 0.05) (Table 1).

**TABLE 1:** Baseline information of included patients with placenta previa accreta.

	Massive hemorrhage group (*n* = 176)	Non-massive hemorrhage (n = 199)	t/χ ^2^	*P*
Maternal age (years)*	32.95 ± 4.53	32.38 ± 4.31	1.27	0.207
BMI (kg/m^2^)*	26.79 ± 3.39	26.07 ± 2.60	−2.27	0.024
Parity*	2.05 ± 0.63	1.87 ± 0.50	−3.01	0.003
Abortion history (time)^#^	2 (1–3)	2 (1–3)	−0.36	0.719
Previous cesarean section (time)*	1.09 ± 0.30	1.13 ± 0.45	−0.88	0.379
HGB (g/L)*	114.27 ± 12.85	112.65 ± 13.45	−1.19	0.236
HCT*	34.53 ± 3.33	33.91 ± 3.70	−1.70	0.090
PLT (*10^9^/L) ^#^	170.00 (141.00–207.00)	166.00 (134.00–206.00)	−0.80	0.452
PT (s)*	11.65 ± 0.75	11.77 ± 0.79	1.46	0.146
APTT (s)*	28.11 ± 3.43	28.54 ± 3.71	1.14	0.254
FIB (mg/dl)*	401.33 ± 74.04	401.58 ± 80.72	0.03	0.975
TT (s)*	16.53 ± 0.63	16.56 ± 0.72	0.52	0.601
Placenta increta/percreta^ &^	170 (96.59)	103 (51.76)	94.80	0.000
Uterine artery embolization^ &^	109 (61.93)	79 (39.70)	18.47	0.000
Gestational weeks of delivery*	37.11 ± 1.09	35.95 ± 0.87	−11.38	0.000

*present with mean ± SD, ^#^present with median (interquartile range), ^&^present with numbers (percentage). BMI, body mass index; HGB, hemoglobin; HCT, hematocrit; PLT, platelets; PT, prothrombin time; APTT, activated partial thromboplastin time; FIB, fibrinogen; TT, thrombin time.

Multivariate logistic regression analysis on risk factors showed that placental increta/percreta and term delivery were independent risk factors for massive hemorrhage in patients with placenta previa accreta. The risk of massive hemorrhage in patients with placenta increta/percreta was 13.65 times that in patients without placenta increta/percreta. Pregnancies delivered after 37 weeks of gestation had 5.00 times as much massive hemorrhage as pregnancies delivered before 37 weeks of gestation (Supplementary Table 1).

### Perioperative period hemodynamics in patients with placental previa accreta

**FIGURE 2:**
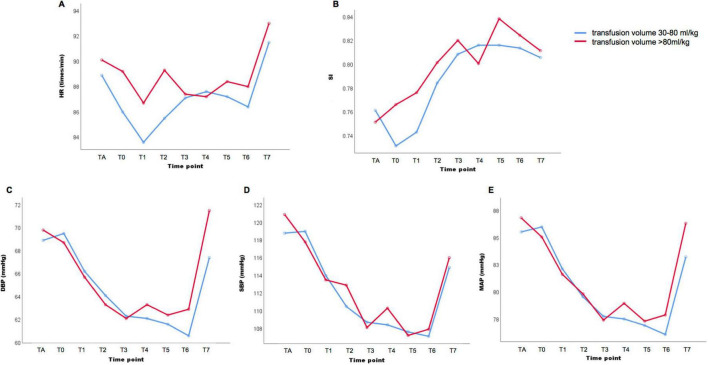
Hemodynamics indicators in patients diagnosed with placenta previa accreta and received transfusion volume 30–80 ml/kg or ≥ 80 ml/kg Maternal heart rate (HR) **(A)**, shock index (SI) **(B)**, diastolic blood pressure (DBP) **(C)**, systolic blood pressure (SBP) **(D)** and mean arterial pressure (MAP) **(E)** in nine different time point: TA, after administration of anesthesia; T0, instantly after baby delivered; T1, 5 min after baby delivered; T2, 10 min after baby delivered; T3, 15 min after baby delivered; T4, 20 min after baby delivered; T5, 25 min after baby delivered; T6, 30 min after baby delivered; T7, the end of procedures.

We divided the patients into three groups according to the volume of intravenous fluid supplement administered during the perioperative period. Group A received a transfusion volume of < 30 ml/kg (n = 53); group B received a transfusion volume of 30–80 ml/kg (n = 270), and group C received a transfusion volume of ≥ 80 ml/kg (n = 52). The hemodynamic indicators, maternal heart rate (HR), SBP, DBP, MAP, and SI, at nine time points from the administration of anesthesia until the end of the procedures, were analyzed among different groups and at different time points. Analysis of variance for repeated measurement analysis demonstrated significant differences among the nine time points in HR, SBP, DBP, MAP, and SI levels (*P* < 0.001). No statistically significant differences were found in maternal HR, SBP, DBP, MAP, or SI among the three different transfusion volume groups (*P* > 0.05). The average values of maternal SBP, DBP, and MAP were 106–122 mmHg, 62–71 mmHg, and 77–88 mmHg, respectively (Supplementary Figure 1 and Supplementary Table 2). Meanwhile, all patients with severe PPH received transfusion volumes higher than 30 ml/kg, and the expression trend of hemodynamic indicators in patients with severe PPH showed no statistical difference between the transfusion volumes of the 30–80 ml/kg and ≥ 80 ml/kg groups (*P* > 0.05) (Figure 2). Maternal SBP, DBP, and MAP started to decrease after the administration of anesthesia and reached a relatively stable state from 15 min to 30 min after delivery (Table 2). Subgroup analysis in patients with placenta increta/percreta, blood loss volume of > 2000 ml, and hysterectomy showed significant differences among the nine time points for maternal HR, SBP, DBP, MAP, and SI values in all subgroup analyses (*P* < 0.001), and no statistical difference was found among different transfusion volume groups in subgroup analysis (Supplementary Table 3).

**TABLE 2:** Hemodynamics indicators in patients with placenta previa accreta and transfusion volume ≥ 30ml/kg.

	TA	T0	T1	T2	T3	T4	T5	T6	T7	F	*P*
HR (beat/min)	91.60 ± 1.06	89.49 ± 1.14	87.18 ± 1.00	88.21 ± 0.97	87.44 ± 1.13	86.89 ± 0.99	88.21 ± 1.01	89.33 ± 1.00	93.75 ± 1.18	10.88	< 0.001
SBP (mmHg)	122.08 ± 1.13	118.94 ± 1.14	114.14 ± 1.03	110.55 ± 1.10	107.82 ± 1.20	108.17 ± 1.16	106.38 ± 1.17	106.15 ± 1.09	116.89 ± 1.07	47.61	< 0.001
DBP (mmHg)	70.98 ± 0.81	70.20 ± 0.91	66.50 ± 0.78	63.95 ± 0.83	62.97 ± 0.89	62.91 ± 0.87	62.52 ± 0.75	62.32 ± 0.85	71.18 ± 0.87	36.26	< 0.001
MAP (mmHg)	88.05 ± 0.81	86.49 ± 0.89	82.40 ± 0.78	79.48 ± 0.86	77.84 ± 0.91	77.92 ± 0.90	77.17 ± 0.80	77.01 ± 0.84	86.19 ± 0.85	48.29	< 0.001
SI	0.76 ± 0.01	0.76 ± 0.01	0.78 ± 0.01	0.81 ± 0.01	0.83 ± 0.01	0.82 ± 0.01	0.85 ± 0.01	0.86 ± 0.01	0.81 ± 0.01	16.22	< 0.001

HR, maternal heart rate; SBP, systolic blood pressure; DBP, diastolic blood pressure; MAP, mean arterial pressure; SI, shock index. Time point: TA, after administration of anesthesia; T0, instantly after baby delivered; T1, 5 min after baby delivered; T2, 10 min after baby delivered; T3, 15 min after baby delivered; T4, 20 min after baby delivered; T5, 25 min after baby delivered; T6, 30 min after baby delivered; T7, the end of procedures.

### Maternal and neonatal outcomes

The mean gestational weeks of delivery in group C were significantly later than those in groups B and A, and those in group B were significantly later than those in group A (*P* < 0.05). One-third of the patients in group A underwent an emergency cesarean section due to indications of antenatal hemorrhage or fetal factors, which was significantly higher than that in groups B and C (*P* < 0.05). More than half of the patients in groups B and C used interventional radiology to reduce uterine bleeding, which was significantly higher than that in group A (*P* < 0.05). The majority of the included patients with placenta previa accreta underwent the procedure under general anesthesia. 17.04% of patients in group B underwent hysterectomy, compared to 55.77% in group C (*P* < 0.05). Although the median procedure time in group C [164 min (Inter quartile range (135–204 min)] was significantly longer than that in groups B and A (*P* < 0.05), the in-hospital days after the procedure demonstrated no statistical differences among the three groups (*P* > 0.05). 96.23% of patients in group A had a blood loss volume of < 1500 ml, while 92.31% of patients in group C had a blood loss volume of ≥ 2000 ml, indicating that the blood loss volume in group B is significantly higher than that in group A and even higher in group C (*P* < 0.05). More than half of the patients in group B received blood transfusions, and all patients in group C received blood transfusions (*P* < 0.05). The urine volume in group A was lower than that in group B, and that in group B was lower than that in group C (*P* < 0.05). Hypotension was observed in 30.19% to 44.23% of patients with placenta previa accreta; no differences were found among the three groups (*P* > 0.05). SI ≥ 1 at three or more continuous time points demonstrated no significant difference among the three groups (*P* > 0.05). The ratios of neonatal mild asphyxia and NICU admission in group C were slightly higher than those in groups A and B; no statistical differences were found among the three groups (*P* > 0.05) (Table 3). Only one neonate in Group B had severe asphyxia.

**TABLE 3:** Maternal and fetal outcomes of patients with placenta previa accreta among different transfusion volume groups.

	Group A (*n* = 53)	Group B (*n* = 270)	Group C (*n* = 52)	Statistic	*P*
Gestational weeks of delivery*	35.59 ± 1.16	36.46 ± 0.87	37.59 ± 1.44	52.16	0.000^(^
Emergency CS^&^	16 (30.19)	40 (14.81)	11 (21.15)	7.58	0.023^£^
Interventional radiology^&^	15 (28.30)	141 (52.22)	33 (63.46)	14.26	0.001^§^
General anesthesia^&^	47 (88.68)	242 (89.63)	45 (86.54)	0.44	0.804
Hysterectomy^&^	-	46 (17.04)	29 (55.77)	56.31	0.000
Procedure time (min)^#^	63 (45–75)	87 (61–124)	164 (135–204)	98.97	0.000^(^
In-hospital days after procedure (d)^#^	6 (4–9)	6 (4–7)	6 (5–7)	2.82	0.244
Blood loss volume (ml)^#^	700 (500–1000)	1300 (800–2050)	4070 (2900–5071)	131.55	0.000^(^
<1500 ml^&^	51 (96.23)	146 (54.07)	2 (3.85)	90.33	0.000^(^
1500–2000 ml^&^	2 (3.77)	42 (15.56)	2 (3.85)	9.69	0.008^£^
≥ 2000 ml^&^	-	82 (30.37)	48 (92.31)	69.48	0.000
Blood transfusion *n* (%)^&^	10 (18.87)	154 (57.04)	52 (100.00)	70.87	0.000^(^
Urine volume (ml)^#^	200 (100–300)	400 (200–600)	1350 (750–2000)	117.03	0.000^(^
Hypotension *n* (%)^&^	16 (30.19)	97 (35.93)	23 (44.23)	2.29	0.136
SI ≥ 1 *n* (%)^&▲^	13 (24.53)	37 (13.70)	9 (17.31)	4.03	0.133
Mild asphyxia *n* (%)^&^	3 (5.66)	18 (6.67)	6 (11.54)	3.03	0.521
NICU admission *n* (%)^&^	10 (18.87)	47 (17.41)	16 (30.77)	4.98	0.083

group A, transfusion volume < 30ml/kg; group B, transfusion volume 30–80 ml/kg; group C transfusion volume ≥ 80 ml/kg. *present with mean ± SD, ^#^present with median (interquartile range), ^&^present with numbers (percentage). CS, cesarean section; SI, shock index; NICU, neonatal intensive care unit. ^(^ difference was found among group A, B and C, statistical differences were found between group A and group B, group B and group C, group A and group C. ^£^difference were found among group A, B and C, statistical differences were found between group A and group B, group B and group C. ^§^ difference were found among group A, B and C, statistical differences were found between group A and group B, group A and group C. ^▲^SI ≥ 1 in continuous three or more-time points were calculated.

## Discussion

Placenta increta/percreta is an independent risk factor for massive hemorrhage in patients with placenta previa accreta, and this risk significantly increases when the gestational age at delivery is more than 37 weeks. A fluid supply volume of 30–80 ml/kg could satisfactorily maintain the stability of maternal hemodynamics during cesarean section, hemostatic operations, and even hysterectomy in patients with placenta previa accreta, considering that no statistical differences were found in hemodynamic indicators or in-hospital days after procedures among the different volume resuscitation groups. Since the maternal SBP, DBP, and MAP started to decrease after the administration of anesthesia and reached a relatively stable low level from 15 to 30 min after delivery, this period is the critical stage for fluid resuscitation in patients with placenta previa accreta.

The overall incidence of placenta previa in this four-and-a-half-year clinical cohort was approximately 1.33%, compared to 0.56 % reported in the literature (25). This discrepancy is partly ascribed to the lack of standardized imaging criteria for placenta previa and inconsistent standards. This retrospective cohort study included patients diagnosed with placenta previa accreta by antenatal ultrasonography and MRI in combination with intraoperative examination and postoperative placental histopathological detection. In this cohort, approximately 20% (75/375) women diagnosed with placenta previa accreta had caesarean hysterectomy. This ratio is similar with another published study, which reported a caesarean hysterectomy rate of 17.4% (326/1874) in women with placenta previa/accreta and complicated with postpartum hemorrhage (26). Another research included and compared several studies on hysterectomy rate, which is ranged between 13–48.9%, in women complicated with placenta previa accreta (27). Additionally, the risk of massive hemorrhage increases rapidly after 36 weeks of gestation (5), and a scheduled birth by caesarean section at 36–37 weeks of gestation is recommended in women with uncomplicated placenta previa (28). In this cohort, the higher ratio of massive hemorrhage in women delivery later than 37 weeks of gestation might be due to the increase uterine activity or emergency surgery. Other potential reason need to be further explored in more studies.

Fluid management is a crucial component of PPH treatment to guarantee adequate perfusion of the vital organs. Both fluid overload and de-resuscitation are associated with adverse maternal outcomes: fluid overload is associated with dilutive coagulopathy and hemorrhage aggravation, and de-resuscitation might lead to hemodynamic instability and poor organ perfusion (29). Parturient women may present with cardiovascular stability even when there is significant blood loss due to a physiological increase in blood volume and cardiac output (12, 30). In pregnant women, the mean MAP between 34 and 37 weeks of gestation is approximately 84–86 mmHg (15), which is significantly higher than that in the first trimester of pregnancy (30). Proper fluid management for patients with placenta previa accreta during the perioperative period requires the participation of obstetricians and anesthetists. The fluid supply volume should be determined based on the speed and volume of blood loss, the estimated time needed for subsequent procedures, and hemodynamic conditions. In an open-label randomized controlled trial, no differences were found in the need for blood transfusion, coagulation parameters, or adverse events between the restrictive and liberal fluid resuscitation groups in patients with mild obstetric hemorrhage (31). According to this retrospective cohort, in patients diagnosed with placenta previa accreta, when the estimated blood loss volume is < 1500 ml, the fluid supply volume is mostly limited to < 30 ml/kg; for parturients with an estimated blood loss volume ≥ 2000 ml, the fluid supply volume could be controlled at 30–80 ml/kg; and in patients complicated by placenta increta/percreta or hysterectomy, the fluid supply volume usually reaches > 80 ml/kg depending on the actual situation. Restrictive fluid resuscitation may be effective in maintaining hemodynamic stability in patients with placenta previa accreta, even if blood loss ≥ 2000 ml or hysterectomy is inevitable. Furthermore, maternal SBP, DBP, and MAP started to decrease immediately after the baby was delivered and reached a relatively stable trough state from 15 min to 30 min after delivery in all included patients with placenta previa accreta. A time period of 15–30 min after delivery is the critical stage for fluid resuscitation.

Patients complicated by an abnormally lying or invasive placenta need to schedule a planned cesarean section after balancing the risk of preterm birth in neonates and massive hemorrhage in mothers to achieve optimal outcomes (9). The RCOG guidelines recommend late preterm (34^+0^ to 36^+6^ weeks of gestation) delivery for women presenting with placenta previa accreta and a history of vaginal bleeding or other associated risk factors for preterm delivery; for women presenting with uncomplicated placenta previa, delivery should be considered between 36^+0^ and 37^+0^ weeks of gestation. In women with placenta accreta spectrum without risk factors for preterm delivery, planned delivery at 35^+0^–36^+6^ weeks of gestation provides the best balance between fetal maturity and the risk of an unscheduled delivery (5). In this retrospective cohort, approximately 30.4% of patients with placenta previa accreta delivered after 37 weeks of gestation, which had a dramatically higher risk of massive hemorrhage. Possible reasons for delayed delivery include the following: Our hospital is the referral center for the southwest areas of China, and a large proportion of the included patients are referred from rural villages and towns. The patient was admitted to our hospital after 37 weeks of gestation. This reflects the insufficient recognition of clinicians in remote community medical institutions regarding the antenatal diagnosis of abnormally lying or invasive placentas as well as the optimal delivery time in patients suspected of placenta previa accreta. It is relatively difficult to diagnose placenta previa accreta and concurrent placenta increta/percreta. Early identification of abnormally lying and invasive placenta is crucial in formulating clinical management to improve perinatal outcomes (5–8).

This study has several strengths. The sample size of patients diagnosed with placenta previa accreta was relatively large. We collected complete hemodynamic data from the administration of anesthesia until the end of the surgical procedure. Our results provide evidence for a restrictive fluid resuscitation strategy in patients with placenta previa accreta, who are at a high risk of massive hemorrhage. This study has several limitations. First, the hemodynamic indicators were monitored using a noninvasive method, in which over 80% of patients performed the procedure under general anesthesia, and the other patients underwent intraspinal anesthesia (epidural anesthesia or combined spinal-epidural anesthesia). Hemodynamic indicators might be influenced by the anesthesia method; we could not perform subgroup analysis due to the small sample size in patients with intraspinal anesthesia. Second, we analyzed the total volume of fluid management rather than the speed of fluid supply, considering the speed of fluid supply had individual difference according to the judgment made by obstetricians and anesthetists. The fluid supply speed in the active obstetric hemorrhage phase is also a vital element in guaranteeing the stability of hemodynamics and saving parturients’ lives. Third, as a retrospective cohort study, we are unable to get the information of placental score values from the ultrasound in most of the cases. Further studies with prospective design is needed to provide more evidence.

Complications with placenta increta/percreta and gestational age of delivery later than 37 weeks are two independent risk factors of massive hemorrhage in patients with placenta previa accreta. Restrictive fluid resuscitation could achieve good effects in maintaining hemodynamic stability during cesarean section, hemostatic operation and even hysterectomy in patients with placenta previa accreta, even in the case of blood loss ≥ of 2000 ml or hysterectomy, which is inevitable. A time period of 15–30 min after baby delivered is the critical stage for fluid resuscitation. Planned delivery should be arranged in patients with placenta previa accrete before 37 weeks of gestation, and the ability to identify concurrent placenta increta/percreta should be improved in order to reduce the adverse perinatal outcomes.

## Data Availability

The raw data supporting the conclusions of this article will be made available by the authors, without undue reservation.
