# Supplementary figures and images for: Fluid resuscitation strategy in patients with placenta previa accreta: a retrospective study
Source: Front Med (Lausanne). 2024 Sep 24;11:1454067. doi: 10.3389/fmed.2024.1454067 (PMC11458411; doi:10.3389/fmed.2024.1454067)

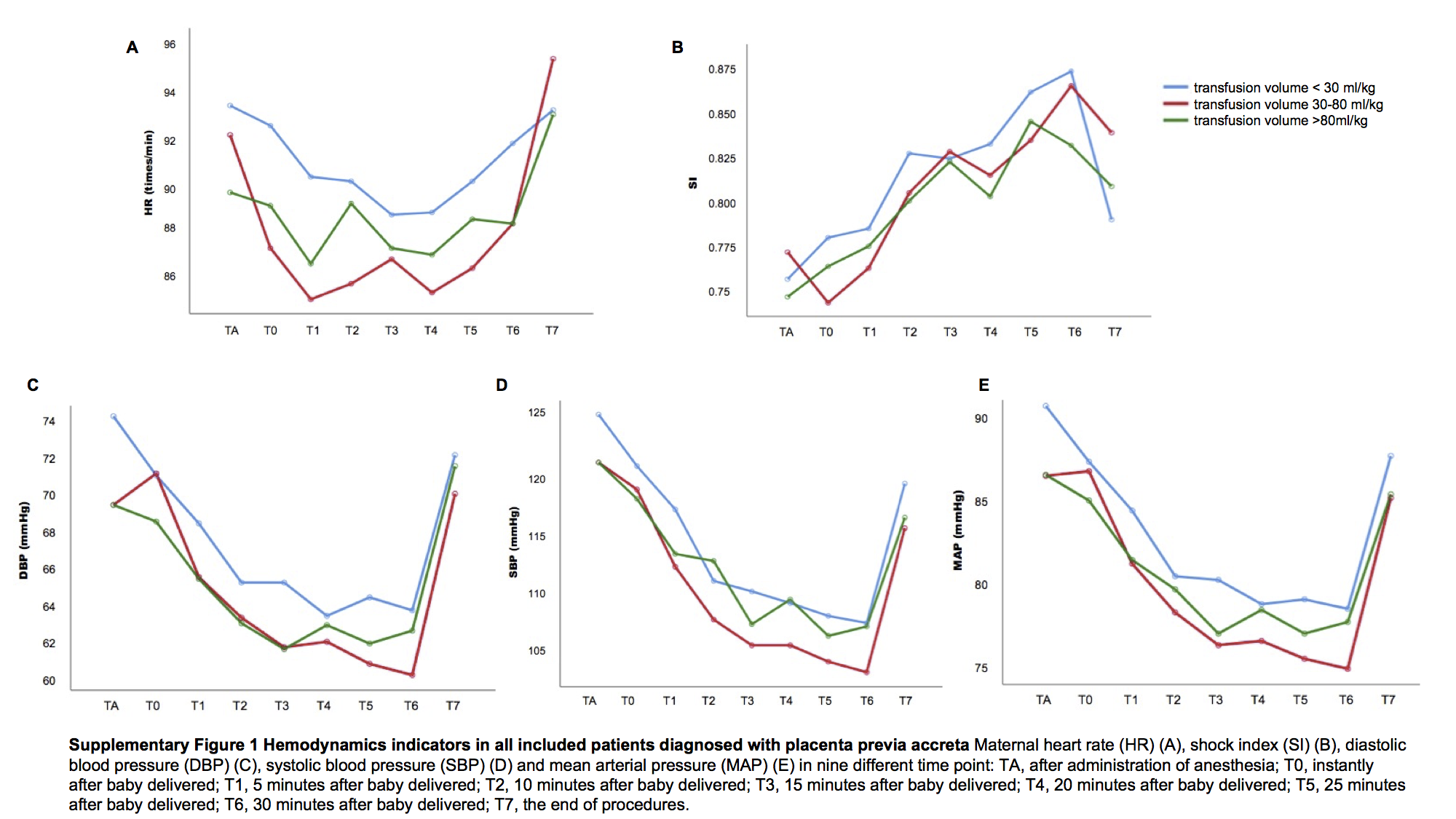

Supplement: Supplementary file 1 [file Image_1.TIFF]
