# Supplementary material for: Fluid resuscitation strategy in patients with placenta previa accreta: a retrospective study
Source: Front Med (Lausanne). 2024 Sep 24;11:1454067. doi: 10.3389/fmed.2024.1454067 (PMC11458411; doi:10.3389/fmed.2024.1454067)
Supplement: Supplementary file 2 [file Table_1.docx]

**Supplementary materials**

**Supplementary table 1 Multivariate logistic regression analysis of risk factors for massive hemorrhage in patients with placenta previa accreta**

|  | **unstandardized coefficients b** | **standard error** | **Wald**  **statistic** | ***P*** | **OR (95% CI)** |
| --- | --- | --- | --- | --- | --- |
| maternal age (years) | -0.333 | 0.331 | 1.016 | 0.314 | 0.716 (0.375, 1.370) |
| BMI ≥ 28kg/m^2^ | 0.037 | 0.327 | 0.013 | 0.910 | 0.964 (0.508, 1.828) |
| parity ≥ 2 | -0.089 | 0.533 | 0.028 | 0.867 | 0.915(0.322, 2.598) |
| abortion ≥ 2 | 0.387 | 0.434 | 0.795 | 0.373 | 1.473 (0.629, 3.451) |
| previous CS ≥ 2 | 0.120 | 0.506 | 0.056 | 0.812 | 1.128 (0.418, 3.041) |
| placenta increta/percreta | 2.766 | 0.509 | 26.323 | 0.000 | 13.651 (5.029, 37.052) |
| gestational age of delivery ≥37 weeks | 1.609 | 0.501 | 10.300 | 0.001 | 4.997 (1.871, 13.346) |
| emergency CS | -0.629 | 0.418 | 2.270 | 0.079 | 0.533 (0.235, 1.208) |

Note: BMI, body mass index; CS, cesarean section.

**Supplementary table 2 Hemodynamics indicators in patients with placenta previa accreta**

|  | **transfusion volumes** | **TA** | **T0** | **T1** | **T2** | **T3** | **T4** | **T5** | **T6** | **T7** | ***F*** | ***P*** |
| --- | --- | --- | --- | --- | --- | --- | --- | --- | --- | --- | --- | --- |
| HR (beat/min) | < 30ml/kg | 93.59±14.46 | 92.55±14.70 | 90.57±14.13 | 90.09±13.68 | 88.77±14.24 | 88.87±14.63 | 90.38±16.52 | 91.98±15.30 | 93.21±14.51 | 2.21 | 0.060 |
|  | 30~80 ml/kg | 90.87±14.44 | 87.09±14.75 | 84.96±13.79 | 84.70±14.64 | 85.81±14.01 | 85.83±13.75 | 85.52±13.76 | 92.33±14.43 | 85.65±13.93 | 27.36 | 0.000 |
|  | ≥ 80 ml/kg | 89.52±16.25 | 88.94±14.60 | 86.44±15.41 | 89.04±15.87 | 87.12±15.38 | 86.83±14.45 | 88.37±14.84 | 88.17±13.32 | 92.89±17.56 | 2.57 | 0.028 |
| SBP (mmHg) | < 30ml/kg | 124.43±15.28 | 120.09±17.05 | 116.60±15.25 | 110.85±15.21 | 110.00±15.50 | 109.06±14.45 | 107.93±17.85 | 107.64±15.24 | 118.87±12.96 | 18.46 | 0.000 |
|  | 30~80 ml/kg | 121.96±15.02 | 119.24±15.10 | 113.63±13.97 | 110.00±13.64 | 107.80±13.64 | 107.91±13.21 | 107.15±13.17 | 116.02±14.11 | 106.98±13.79 | 86.64 | 0.000 |
|  | ≥ 80 ml/kg | 120.77±16.07 | 117.79±13.11 | 113.27±14.55 | 112.69±15.10 | 107.50±15.67 | 109.52±16.25 | 106.54±15.07 | 107.31±13.67 | 116.25±14.98 | 4.47 | 0.001 |
| DBP (mmHg) | < 30ml/kg | 74.06±11.31 | 71.13±12.23 | 68.30±11.80 | 65.09±10.85 | 65.19±11.31 | 63.49±8.69 | 64.43±11.50 | 63.96±11.15 | 72.08±11.33 | 11.71 | 0.000 |
|  | 30~80 ml/kg | 71.17±10.42 | 66.54±10.55 | 71.54±11.63 | 63.78±10.10 | 62.35±10.19 | 62.09±9.43 | 62.20±9.70 | 61.87±9.55 | 69.87±11.46 | 84.08 | 0.000 |
|  | ≥ 80 ml/kg | 69.62 ±9.79 | 68.75±10.57 | 65.58±10.08 | 63.17±9.90 | 61.73±11.75 | 62.88±11.43 | 62.21±10.82 | 62.88±11.60 | 71.44±11.22 | 4.81 | 0.000 |
| MAP (mmHg) | < 30ml/kg | 90.85±11.56 | 87.45±12.92 | 84.40±12.06 | 80.35±11.55 | 80.13±11.80 | 78.68±9.94 | 78.93±13.02 | 78.52±11.61 | 87.67±11.11 | 16.51 | 0.000 |
|  | 30~80 ml/kg | 88.10±10.89 | 87.44±11.79 | 82.24±10.91 | 79.19±10.64 | 77.50±10.61 | 77.36±10.01 | 77.19±10.12 | 77.19±10.12 | 85.25±11.03 | 105.21 | 0.000 |
|  | ≥ 80 ml/kg | 86.67±10.67 | 85.10±10.01 | 81.47±10.40 | 79.68±10.74 | 76.99±11.99 | 78.43±12.18 | 76.99±11.05 | 77.69±11.45 | 85.48±12.41 | 11.94 | 0.000 |
| SI | < 30ml/kg | 0.76±0.14 | 0.78±0.16 | 0.79±0.17 | 0.83±0.18 | 0.83±0.20 | 0.84±0.21 | 0.87±0.25 | 0.87±0.21 | 0.79±0.14 | 5.41 | 0.001 |
|  | 30~80 ml/kg | 0.76±0.14 | 0.74±0.14 | 0.76±0.15 | 0.78±0.16 | 0.81±0.15 | 0.81±0.15 | 0.81±0.14 | 0.81±0.16 | 0.80±0.14 | 14.76 | 0.000 |
|  | ≥ 80 ml/kg | 0.75±0.13 | 0.76±0.15 | 0.78±0.17 | 0.80±0.17 | 0.82±0.16 | 0.80±0.14 | 0.84±0.18 | 0.83±0.15 | 0.81±0.17 | 4.29 | 0.001 |

Note: HR, maternal heart rate; SBP, systolic blood pressure; DBP, diastolic blood pressure; MAP, mean arterial pressure; SI, shock index. Time poing: TA, after administration of anesthesia; T0, instantly after baby delivered; T1, 5 minutes after baby delivered; T2, 10 minutes after baby delivered; T3, 15 minutes after baby delivered; T4, 20 minutes after baby delivered; T5, 25 minutes after baby delivered; T6, 30 minutes after baby delivered; T7, the end of procedures.

**Supplementary Table 3 Repeated measurement ANOVA analysis of hemodynamics indicators in patients with placenta previa accreta**

| hemodynamics indicators | **time point** | | | **groups** | | | **groups * time point** | |
| --- | --- | --- | --- | --- | --- | --- | --- | --- |
|  | ***F*** | ***P*** | | ***F*** | ***P*** | | ***F*** | ***P*** |
| HR (beat/min) | 10.88 | | ＜0.001 | 0.69 | | 0.505 | 1.36 | 0.207 |
| SBP (mmHg) | 47.61 | | ＜0.001 | 1.49 | | 0.231 | 0.63 | 0.749 |
| DBP (mmHg) | 36.26 | | ＜0.001 | 1.76 | | 0.177 | 0.86 | 0.556 |
| MAP (mmHg) | 48.29 | | ＜0.001 | 1.86 | | 0.162 | 0.66 | 0.729 |
| SI | 16.22 | | ＜0.001 | 0.14 | | 0.869 | 0.96 | 0.466 |

Note: HR, maternal heart rate; SBP, systolic blood pressure; DBP, diastolic blood pressure; MAP, mean arterial pressure; SI, shock index.
